# Is Semaglutide Linked to NAION? A Case Report on a Rare Ocular Complication

**DOI:** 10.3390/reports8030149

**Published:** 2025-08-20

**Authors:** Dina Lešin Gaćina, Tomislav Vidović, Nikolina Vlajić Oreb, Lovorka Matković, Sonja Jandroković

**Affiliations:** 1 Department of Ophthalmology, Zagreb University Hospital Center, 10 000 Zagreb, Croatia; tvidovic@kbc-zagreb.hr (T.V.); sjandrokovic@mef.hr (S.J.); 2 Vuk Vrhovac University Clinic for Diabetes, Endocrinology and Metabolic Diseases, Merkur University Hospital, 10 000 Zagreb, Croatia; vlajic.nina@gmail.com; 3 Medical Center of Zagreb County, 10 430 Samobor, Croatia; lovorkamatkovic@yahoo.com; 4 School of Medicine, University of Zagreb, 10 000 Zagreb, Croatia

**Keywords:** ischemic optic neuropathy, diabetes mellitus, semaglutide, GLP-1 receptor agonists, case report

## Abstract

**Background and Clinical Significance**: Ischemic optic neuropathies (IONs) are significant causes of vision loss resulting from compromised blood flow to the optic nerve. Diabetes mellitus (DM) exacerbates the risk of IONs through chronic hyperglycemia and associated vascular dysfunction. Recently, semaglutide, a glucagon-like peptide-1 (GLP-1) receptor agonist, has been linked to ocular complications, including non-arteritic anterior ischemic optic neuropathy (NAION), potentially due to the rapid glycemic changes or vascular effects. **Case Presentation**: A 55-year-old female with type 2 DM, hypertension, and hyperlipidemia presented with blurred vision and optic disc edema after four months of semaglutide therapy (Ozempic^®^, Sydney, Australia). Initially diagnosed with diabetic papillopathy (DP), her condition progressed to NAION, leading to partial visual recovery with corticosteroid treatment and improved glycemic management. Diagnostic evaluations, including visual field testing, optical coherence tomography, and fluorescein angiography, supported the diagnosis. **Conclusions**: This case report describes the clinical course of a diabetic patient treated with a semaglutide who developed an ischemic optic event. The timing of symptom onset in relation to the initiation of semaglutide therapy raises the possibility of a causal association between the drug and this rare ocular complication. Close monitoring of ocular health is crucial for patients on GLP-1 receptor agonists, particularly those with pre-existing vascular risk factors. Further research is needed to elucidate the underlying mechanisms and guide clinical practice.

## 1. Introduction and Clinical Significance

Ischemic optic neuropathies (IONs) are significant causes of vision loss, resulting from compromised blood flow to the optic nerve. This leads to damage to nerve fibres and the potential partial or complete loss of optic nerve function [1]. Diabetes mellitus (DM) is a well-recognized risk factor for IONs, increasing susceptibility through chronic hyperglycemia, vascular inflammation, and a pro-thrombotic state [1]. Chronic hyperglycemia in DM leads to structural changes in blood vessels, decreasing their capacity to supply adequate blood flow to essential tissues, including the optic nerve. Diabetes often coincides with other ION risk factors, such as hypertension, dyslipidemia, and obstructive sleep apnea, which further compromise the perfusion of the optic nerve. These impairments become more significant as the duration and severity of diabetes increase [1].

Diabetic papillopathy (DP), a rare form of diabetes-related ION, presents as optic disc swelling, often without accompanying diabetic retinopathy (DR) [2]. As there are currently no specific diagnostic criteria for DP, it is often considered a diagnosis of exclusion, established only after other potential causes, such as inflammation, infection, neovascularization, papilledema, and optic disc drusen, have been ruled out. DP frequently resolves without treatment but can occasionally progress to mild visual impairment or even more severe ischemic complications, particularly in anatomically predisposed individuals undergoing rapid glycemic improvement [2].

Non-arteritic anterior ischemic optic neuropathy (NAION), the most common form of acute ION in individuals over 50, typically presents as sudden, painless, unilateral vision loss [1]. Risk factors include systemic vascular conditions and a “disc-at-risk” anatomy characterized by a crowded optic nerve head (ONH). The reduced perfusion of small vessels supplying the optic nerve contributes to ischemia, followed by cytotoxic edema and axonal damage, ultimately leading to retinal ganglion cell loss. It is likely that other unknown risk factors also contribute to the development of NAION, as certain medications, such as amiodarone, phosphodiesterase inhibitors like sildenafil, and triptans for migraine treatments, have been associated with its onset [1].

Semaglutide, a glucagon-like peptide-1 (GLP-1) receptor agonist, is widely used as a second-line therapy for type 2 DM and obesity. By mimicking the action of the natural hormone GLP-1, the semaglutide activates pancreatic GLP-1 receptors, enhancing insulin secretion, lowering glucagon levels, slowing gastric emptying, and thereby helping to control blood glucose levels and support weight loss [3]. Clinical trials have demonstrated that the semaglutide reduces cardiovascular risk in diabetic patients, enhancing its role as an important component of comprehensive diabetes management [4]. While GLP-1 agonists show potential neuroprotective effects, findings from the SUSTAIN-6 trial revealed a paradoxical worsening of DR in patients with pre-existing DR, likely due to rapid improvements in glucose control [5]. Building on these findings, a more recent study identified a significant association between semaglutide use and the development of NAION [6]. This case report presents a type 2 DM patient treated with a semaglutide (Ozempic^®^), who developed optic disc edema, initially diagnosed as DP, which later progressed to NAION.

## 2. Case Presentation

A 55-year-old woman presented to the emergency department with a three-day history of blurred, painless vision in the temporal third of her right visual field. The patient’s medical history included type 2 DM, diagnosed six years prior and managed with metformin (1000 mg daily). Four months prior, the patient initiated weekly subcutaneous semaglutide (Ozempic^®^) therapy, starting with 0.5 mg once weekly for four weeks, which was then increased to 1 mg weekly. The treatment was prescribed due to her overweight status, with a body mass index (BMI) of approximately 32. Additional comorbidities included arterial hypertension, controlled using a combination of perindopril and amlodipine, supplemented by nebivolol, and hyperlipidemia, managed with atorvastatin. Her medical history revealed that five years earlier, the patient had experienced an episode of elevated blood glucose levels (approximately 20 mmol/L) following corticosteroid therapy for lumbar spine issues; however, the condition stabilized spontaneously at that time. She was a non-smoker with no history of ocular conditions.

**Figure 1:**
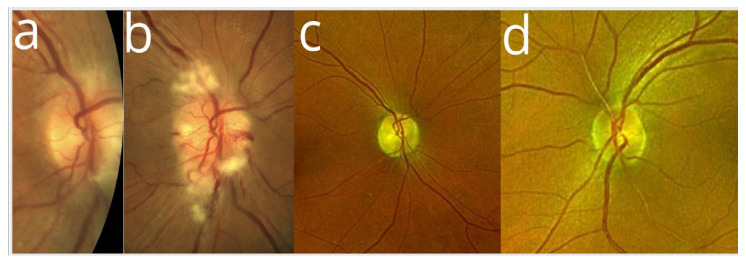
Colour fundus photography presents (**a**) a mild optic disc edema at the superior and inferior poles of the right eye (RE) at initial presentation; (**b**) an active phase of NAION of the RE with visible peripapillary flame-shaped hemorrhages, cotton-wool spots, and optic disc swelling; (**c**) optic disc pallor post-resolution of edema of the RE, indicative of optic atrophy; and (**d**) ONH of the left eye appeared normal in configuration, with a small cup-to-disc ratio.

On examination, her best-corrected visual acuity (BCVA) was 20/20 bilaterally, as assessed using the Snellen chart. At that time, pupillary reactions were normal. A fundus examination revealed indistinct margins of the ONH at the superior and inferior poles of the right eye (Figure 1a). ONH of the left eye appeared with no abnormalities, with a small cup-to-disc ratio (Figure 1d).

**Figure 2:**
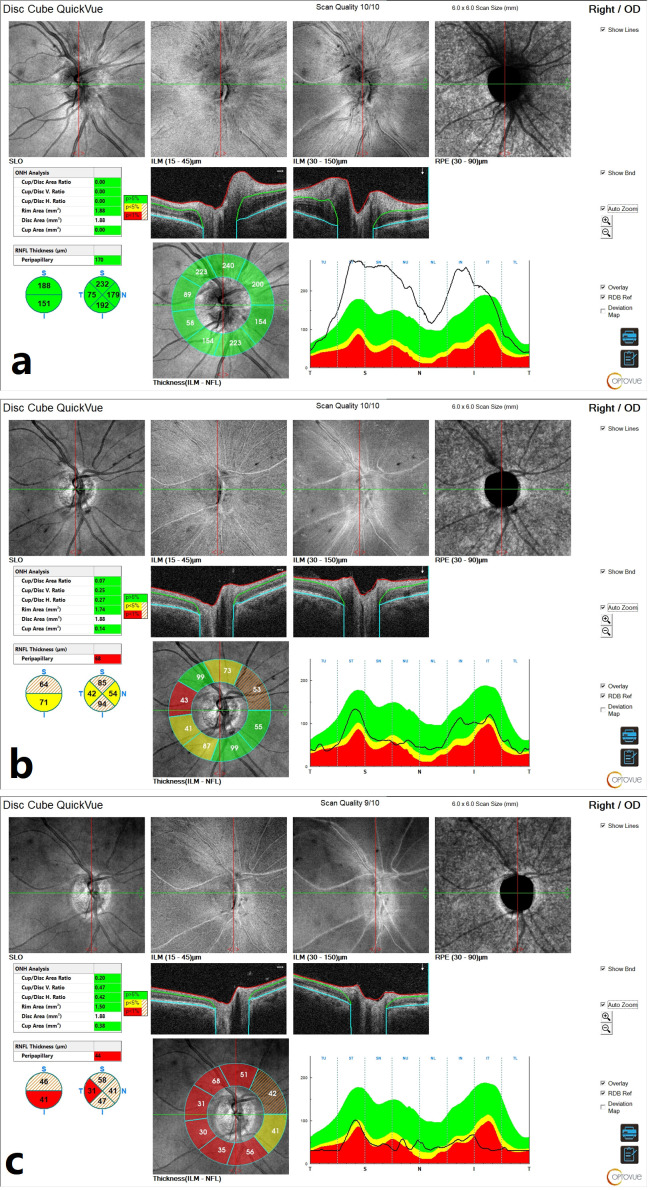
Optical coherence tomography scans of the right eye: (**a**) an increased RNFL thickness at initial presentation, consistent with optic disc edema; (**b**) a gradual resolution of RNFL thickening; and (**c**) progressive RNFL thinning suggestive of optic nerve fibre atrophy during follow-up.

Initial optical coherence tomography (OCT) of the right ONH demonstrated an elevation of the retinal nerve fibre layer (RNFL), consistent with ONH edema (Figure 2a). Macular OCT revealed no abnormalities.

**Figure 3:**
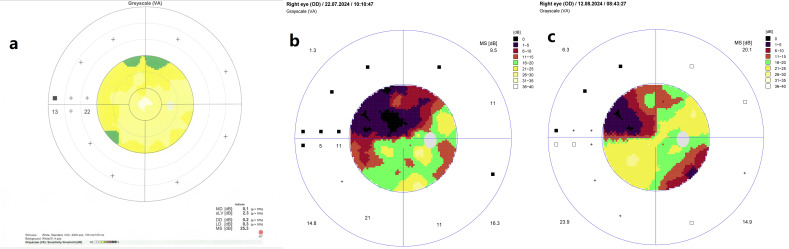
Grey scale visual field plots of the right eye: (**a**) initially preserved retinal sensitivity; (**b**) superior altitudinal defect in the active phase of NAION; and (**c**) partial recovery of the visual field defect during follow-up evaluation.

At that stage, visual field testing using the Octopus 900 G-Program (Dynamic Strategy) showed normal retinal sensitivity (Figure 3a).

**Figure 4:**
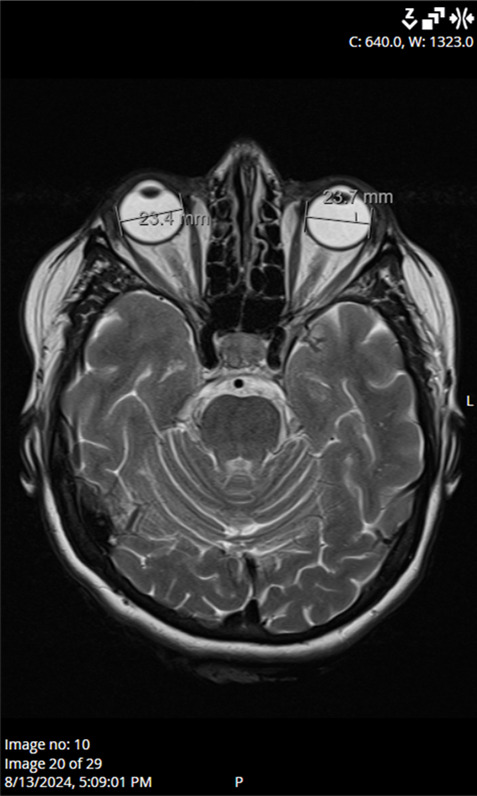
MRI scan of the brain and orbits shows no pathological findings, effectively ruling out an anatomical cause for the optic disc edema.

Ocular ultrasound showed a slight ONH prominence bilaterally, while computed tomography of the brain and orbits showed no pathological findings. Laboratory results, including CRP (6 mg/L), ESR (13 mm/h), platelet levels (251 × 10^9^/L), blood glucose (6.3 mmol/L), and HbA1c levels (5.8%), were within acceptable ranges. Blood pressure, echocardiogram, and Doppler imaging of the carotid and vertebral arteries were unremarkable. Magnetic resonance imaging of the brain and orbits found no abnormalities to explain the optic nerve edema (Figure 4). In addition, antibody testing for aquaporin-4 and myelin oligodendrocyte glycoprotein (MOG) was also performed during hospitalization, and the results returned negative. Serological testing for neurotropic microorganisms revealed negative results.

Based on clinical findings, without significant impairment of visual function, the patient was diagnosed with DP, a rare but typically benign condition associated with diabetes. She was advised to maintain strict control of vascular risk factors and scheduled for follow-up. Two weeks later, the patient returned with worsening vision in her right eye, and she was admitted to the hospital for further diagnostic evaluation.

**Figure 5:**
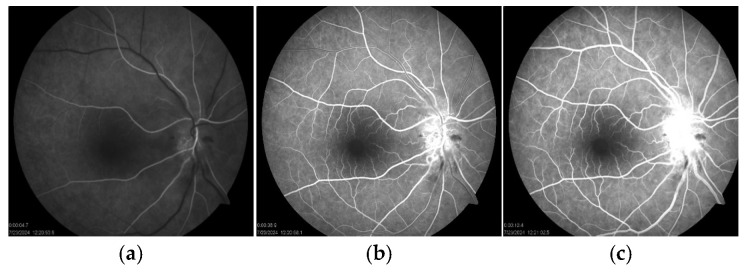
Fluorescein angiographic findings showed (**a**) early arteriovenous phase with juxtapapillary hypofluorescence in the nasal rim, consistent with hemorrhage; (**b**) arteriovenous phase hyperfluorescence highlights peripapillary capillary leakage; and (**c**) late venous phase demonstrates further leakage and impaired microcirculation.

At that time, BCVA had decreased to 20/40, while the left eye remained at 20/20. Examination showed a positive relative afferent pupillary defect and an abnormal Ishihara test in the right eye. A fundus examination showed an optic disc edema with flame-shaped hemorrhages and cotton-wool spots in the peripapillary region (Figure 1b). Fluorescein angiography (FAG) of the right eye demonstrated hyperfluorescence of the optic disc in the late phase, indicative of leakage from dilated peripapillary capillaries and impaired microcirculation (Figure 5a–c).

Moreover, visual field testing revealed a superior altitudinal defect (Figure 3b). Blood tests at this stage showed a mildly elevated C-reactive protein (15.9 mg/L) and a blood glucose level of 8.5 mmol/L. The patient was diagnosed with NAION. In consultation with an endocrinologist, treatment included a three-day course of intravenous methylprednisolone (1 g/day), followed by oral prednisone (75 mg/day) tapered over one month. Blood glucose levels were carefully monitored during steroid therapy, and diabetes management was adjusted by discontinuing the semaglutide, introducing insulin, and increasing metformin to 1000 mg twice daily. At her follow-up visit, the patient’s BCVA in the right eye had improved to 20/20. A fundus examination revealed an optic atrophy in the right eye (Figure 1c). Visual field testing indicated partial recovery of the superior altitudinal defect (Figure 3c), while OCT scans of the ONH showed progressive RNFL thinning, suggestive of optic nerve fibre atrophy during follow-up (Figure 2b,c). The left eye remained unaffected throughout the course of the illness (Figure 1d).

## 3. Discussion

This case report presents the documented clinical course of a patient with obesity and diabetes undergoing treatment with a semaglutide, a GLP-1 receptor agonist, who subsequently developed ION. The temporal association between the initiation of the semaglutide and the onset of NAION suggests a possible link between the medication and this rare ocular complication.

Semaglutide is widely used for glycemic control in type 2 DM and was granted The United States Food and Drug Administration (FDA) approval for long-term weight management in obesity, with proven cardiovascular benefits. This approval has sparked global interest due to its significant short-term weight loss benefits. However, concerns regarding uncritical and potentially unsafe prescribing practices have emerged, raising important health implications. A study by Luo et al. indicates that GLP-1 receptor agonists, particularly semaglutide and lixisenatide, are associated with a notable risk of ocular adverse events based on real-world data [7].

We report the case of an overweight middle-aged woman with type 2 DM who initially presented with unilateral mild optic disc edema in the absence of other ocular abnormalities, along with subtle ophthalmic symptoms. A thorough diagnostic evaluation excluded alternative etiologies, leading to a diagnosis of DP. However, two weeks later, the patient developed clinical features consistent with NAION. The patient’s comorbid conditions, including type 2 DM, hypertension, and hyperlipidemia, are well-established risk factors for vascular dysregulation and ONH ischemia. Interestingly, semaglutide therapy, which had been initiated four months prior to symptom onset, may have acted as a triggering factor in this vulnerable individual. Supporting this association, Karam et al. recently reported a case involving a 73-year-old man who developed bilateral NAION after significant weight loss and postural hypotension, both of which occurred during semaglutide treatment [6].

The current literature lacks sufficient evidence to confirm a causal relationship between GLP-1 receptor agonists and ocular ischemia. However, a retrospective cohort study by Hathaway et al. has highlighted a potential association between semaglutide use and the development of NAION [8]. This study, based on medical registry data, reported an increased absolute risk of NAION of 7.5% in patients with T2DM and 7% in individuals using a semaglutide for obesity. Both groups were found to have a heightened likelihood of developing NAION within 36 months of treatment initiation. Notably, the study indicated that the risk was most pronounced during the first year of semaglutide use. This observation aligns with the timeline in our case. A recent longitudinal study by Grauslund et al. was the first to demonstrate, in a Danish nationwide cohort of individuals with type 2 DM, that the use of once-weekly semaglutide was independently associated with a 2.19-fold increased hazard of developing NAION in five years. In contrast, in their study, the median time to event was 22.2 months following the prescription of a semaglutide [9]. Similar results were reported in a retrospective cohort study by Hsu et al., which found that the use of a semaglutide is associated with an increased risk of NAION in patients with diabetes, particularly after two years of therapy. An increased risk of NAION was also observed in patients with diabetes and concomitant hypertension [10]. On the other hand, a retrospective study conducted by Cai et al. analyzed data from 14 databases, including adults with T2DM who were treated with a semaglutide, other GLP-1 receptor agonists, or non–GLP-1 receptor agonists medications. Their findings suggest a modest increase in the risk of NAION among patients using a semaglutide, although this risk was lower than what has been reported in previous studies [11]. Contrary to the above, the findings of a multicenter retrospective study by Chou et al. suggest that semaglutides may not be associated with an increased risk of NAION in the general population [12]. This highlights the need for further research to clarify this issue.

The exact biological link between semaglutides and ocular ischemic events is still not fully understood, but current evidence suggests the involvement of several interconnected mechanisms. One key factor is the rapid reduction in blood glucose levels, which can lead to retinal hyperperfusion, altered microcirculation, and increased venous pressure [13]. These vascular changes may impair optic nerve perfusion, especially in individuals with pre-existing risk factors or anatomically crowded optic discs (“disc-at-risk”). Similar patterns were observed in the SUSTAIN-6 trial, which reported worsening of DR in patients with a rapid glycemic improvement while using GLP-1 receptor agonists [5]. Although NAION and DR are distinct conditions with different pathophysiological mechanisms, both share a common vascular origin. DR typically involves diffuse microvascular damage throughout the retina, while NAION is a localized ischemic event affecting the ONH. The pathophysiology of NAION resembles compartment syndrome, in which the confined space within the optic nerve restricts blood flow through the posterior ciliary arteries that supply the ONH [1]. Differences in anatomical location, vascular supply, and underlying mechanisms explain the distinct clinical presentations of these two conditions. In this case, the absence of DR may be attributed to the macula’s ability to accommodate extracellular fluid through tissue expansion, a property lacking in the ONH, making the optic nerve more susceptible to ischemic injury.

GLP-1 receptor agonists like the semaglutide also activate endothelial nitric oxide synthase (eNOS), increasing nitric oxide (NO) levels [13]. This typically improves microcirculation through vasodilation, which is beneficial for cardiovascular health. However, in anatomically or physiologically predisposed individuals, this vasodilation may disturb the delicate blood flow balance in the ONH, which has high metabolic demands. In patients with small, crowded optic discs, vasodilation may raise tissue pressure in the confined ONH space, compromising perfusion and leading to localized hypoperfusion. Additionally, excessive vasodilation could trigger a “vascular steal” effect, where blood is diverted away from already susceptible microvascular regions. Increased capillary permeability due to NO may further disrupt local circulation. When combined with systemic risk factors like diabetes and hypertension, these effects may impair autoregulation and heighten the ONH’s susceptibility to ischemic damage.

In our case, the progression from DP to NAION in this case is notable. While DP is generally considered a benign condition, its overlapping clinical features and shared risk factors with NAION complicate accurate diagnosis [14]. The concept of “incipient NAION,” as described by Hayreh and Zimmerman, emphasizes this diagnostic challenge [15]. They reported that approximately 25% of incipient NAION cases, characterized by asymptomatic optic disc edema, progressed to classic NAION, with a median interval of 5.8 weeks. In our patient, the absence of DR and the presence of optic disc edema suggest a transitional phase that may have been misdiagnosed as DP but was likely indicative of early NAION. Although robust clinical evidence is lacking, understanding this relationship is critical. DP may reflect an underlying vascular instability in anatomically predisposed diabetic patients, potentially elevating their risk for ischemic events such as NAION.

## 4. Conclusions

Although anecdotal, this case provides a documented clinical course of a patient with obesity and diabetes who developed NAION following the initiation of semaglutide therapy. While the semaglutide remains a valuable therapeutic option for diabetes and obesity, clinicians should be aware of ischemic eye events as potential side effects. Close monitoring of retinal and optic nerve health is critical, especially in high-risk individuals. Further research is essential to establish causality and elucidate the biological mechanisms linking semaglutide to ischemic ocular events. Longitudinal studies with larger cohorts could provide definitive insights and guide clinical decision-making in the management of patients receiving GLP-1 receptor agonists.

## Data Availability

The data supporting the findings of this study are not publicly available due to the presence of personal information and ethical considerations.

## Abbreviations

The following abbreviations are used in this manuscript:

ION	ischemic optic neuropathies
DM	diabetes mellitus
NAION	non-arteritic anterior ischemic optic neuropathy
GLP-1	glucagon-like peptide-1
BMI	body mass index
DP	diabetic papillopathy
DR	diabetic retinopathy
ONH	optic nerve head
BCVA	best-corrected visual acuity
OCT	optical coherence tomography
FAG	fluorescein angiography
FDA	Food and Drug Administration
e-NOS	endothelial nitric oxide synthase
NO	nitric oxide
